# Supplementary material for: Pretreatment Tumor Growth Rate and Radiological Response as Predictive Markers of Pathological Response and Survival in Patients with Resectable Lung Cancer Treated by Neoadjuvant Treatment
Source: Cancers (Basel). 2023 Aug 17;15(16):4158. doi: 10.3390/cancers15164158 (PMC10453282; doi:10.3390/cancers15164158)
Supplement: Supplementary file 1 [file cancers-15-04158-s001.zip › cancers-2537487 supplementary file.pdf]

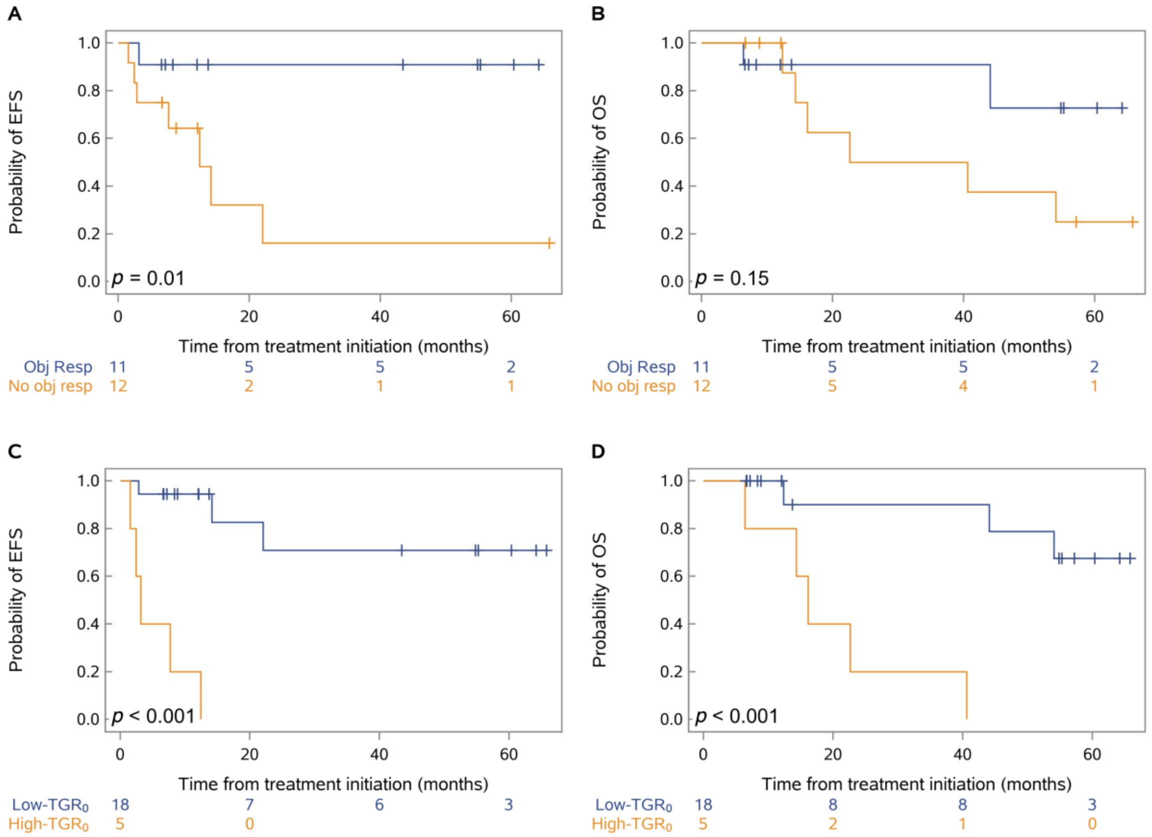

Figure S1. Kaplan–Meier analysis of event-free survival (EFS) and overall survival (OS) by RECIST objective response of the primary tumor (**A**,**B**) and pretreatment TGR0 (**C**,**D**) in a subgroup of patients treated by neoadjuvant nivolumab-based therapy. *p*-values were obtained using the log-rank test.

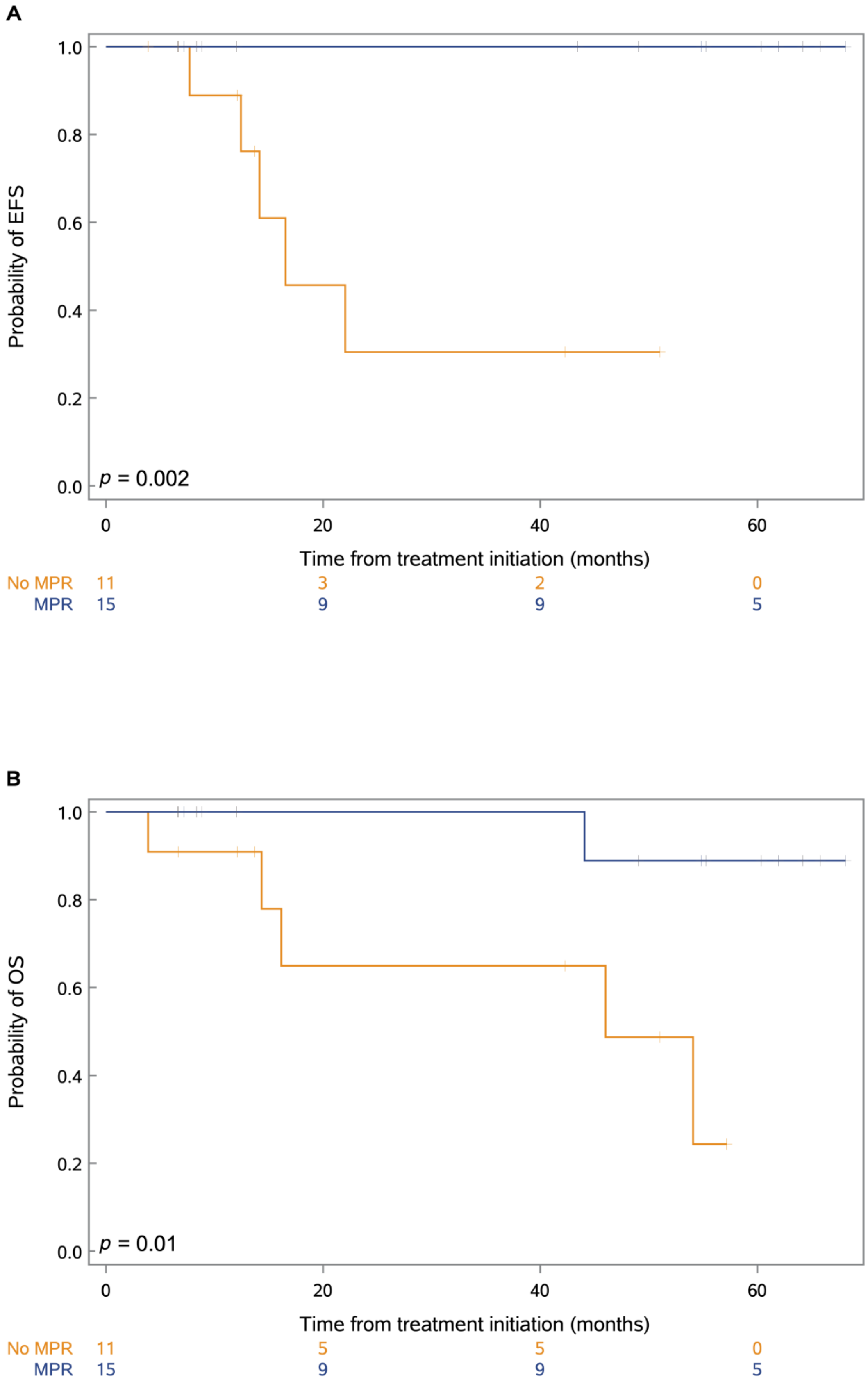

Figure S2. Kaplan–Meier analysis of event-free survival (**A**) and overall survival (**B**) by major pathological response (MPR). *p*-values were obtained using the log-rank test. EFS: event-free survival; OS: overall survival..
